# Impacts of COVID-19 on Young Children and Families: A Qualitative Study Using Best Starts for Kids Health Survey Data in King County, WA

**DOI:** 10.1007/s10995-023-03810-5

**Published:** 2023-11-03

**Authors:** Mohit Nair, Kristin Moore, Sara Jaye Sanford, Anne McNair, Alastair Matheson, Eva Wong

**Affiliations:** 1 Assessment, Policy Development, and Evaluation Unit, Public Health-Seattle & King County, Seattle, USA; 2 Department of Epidemiology, School of Public Health, University of Washington, Seattle, USA

**Keywords:** COVID, Children, Education, Remote, Basic needs, Qualitative

## Abstract

**Objectives:**

To evaluate the impacts of the COVID-19 pandemic on parents and caregivers with young children in King County, Washington using data from a local population-based survey, the Best Starts for Kids Health Survey (BSKHS).

**Methods:**

7033 parents and caregivers in King County, Washington with children 5th grade and younger participated in the BSKHS in 2021. Three evaluators adopted a framework method approach to thematic analysis for open-ended survey responses regarding the impacts of COVID-19 on families.

**Results:**

Children aged 0–5 years missed important social development opportunities, while elementary-school children missed social interactions and felt isolated during remote schooling. Parents were exhausted by the competing demands of work and schooling/childcare, with mothers bearing the brunt of additional responsibilities. Many families faced financial uncertainty and were unable to meet basic needs. Yet, families were grateful for more quality time with family members.

**Conclusions for Practice:**

Parents expressed that children aged 0–5 years missed out on social development opportunities with both adults and children their own age and elementary-school children and felt isolated while schools were closed to in-person schooling.

## Significance

*What is already known on this subject?* The impacts of the COVID-19 pandemic on issues that affect children and families such as employment loss, social isolation, food security, mental health, learning loss, and access to care have been extensively quantitatively documented in MCHJ and elsewhere.

*What does this study add?* This study utilizes critical qualitative data from a racially and linguistically representative population health survey to describe the disproportionate impact of the COVID-19 pandemic on key populations, including but not limited to parents with young children living with disabilities, BIPOC communities, and mothers or female caregivers.

## Background

Starting in March 2020, the coronavirus (COVID-19) pandemic changed the landscape dramatically for children and families. Kindergartens through high schools were mandated to close facilities and offer remote learning starting on March 17, 2020 (Public Health Insider, 2020; Furfaro et al., 2021). In April 2021, schools were required to offer some in-person instruction to all students, and many King County school districts began phasing in hybrid learning at that point (Furfaro et al., 2021). Across King County, an estimated 15,900 children lacked a computer or internet access, disrupting their ability to access remote education (14.8% of students in less affluent suburban areas) (PHSKC, 2021). In the Seattle-Tacoma-Bellevue metropolitan statistical area, about one in ten households with children sometimes or often did not have enough food to eat over the fall and winter of 2020–2021, and almost one in three adults with children experienced childcare unavailability due to COVID-19 (PHSKC, 2021; Schachter et al., 2020).

Prior to the pandemic, children and their families received health, mental health, and developmental supports through a network of services provided by King County and many community-based partners that were primarily offered in-person. Examples include school-based health centers and a program called Early Supports for Infants and Toddlers (ESIT, 2022; King County School Health, 2022). During the pandemic, many of these programs switched to remote services and then hybrid models. Many tried to address barriers that families experienced to remote services by offering telephone interpretation services, internet hotspots, and devices. At the national level, the number of children served by early childhood systems decreased, and programs experienced reduced resources, poor service coordination, and communication challenges between families and providers (CDC, 2022). Among school-aged children, stress and disruption related to the pandemic increased depression and anxiety, leading to increased need for mental health services (Meherali et al., 2021). However, these have not been well-documented at the local level.

This study describes the impacts of the COVID-19 pandemic through September 2021 in King County, Washington (Economic, social, and overall health impacts dashboard, 2022; Race & ethnicity data dashboard, 2021). We used qualitative data to examine overall experiences of families with young children, impacts on emotional health and well-being, childcare and elementary school, family relationships, and disproportionate impacts on Black, Indigenous, and People of Color (BIPOC) communities, since these communities are impacted due to pre-existing and pandemic-era social and structural inequities driven by racism (Tai et al., 2021).

## Methods

### Survey Methods and Analysis

The Best Starts for Kids Health Survey (BSKHS) is a population-based survey about the health and well-being of young children and families in King County, Washington, USA (BSKHS, 2021; Wong et al., 2017). The BSKHS is conducted every two years, and questions about impacts of COVID on children and families were added in 2021. Families with children in 5th grade or younger (6 months–12 years) residing in King County were eligible to participate in the survey. One child per household was selected, with oversampling among smaller regions and racial/ethnic groups. A community-based convenience sample was opened at the end of each survey cycle to increase sample sizes for small populations and to increase community engagement in the survey. Respondents were able to take the survey online or by phone with an interviewer in English, Spanish, Chinese, Vietnamese, Somali, Russian, and Korean.

### Qualitative Methods and Analysis

This study focused specifically on qualitative responses to the following open-ended questions: “*Please describe any changes, positive or negative, that the COVID-19 pandemic has had on this child*” and “*Please describe any changes, positive or negative, that the COVID-19 pandemic has had on your family*.” Participants provided similar responses for the two questions about impact on “this child” and “your family,” so responses were combined for the analysis.

To analyze the data, a framework method approach was adopted for thematic analysis (Gale et al., 2013). This approach offered flexibility in distilling a high volume of responses. Three evaluators (KM, SJS, and MN) independently read through several open-ended responses and familiarized themselves with the data. The team jointly developed a codebook with shared definitions of codes based on a priori knowledge and topics of interest, such as social-emotional and mental health and childcare, among others. The codebook was iteratively adapted following a review of a random sample of open-ended responses, and additional codes were inductively derived, such as employment.

The framework matrix was developed in Microsoft Excel, which allowed for a cross-case analysis and comparisons across codes. Each code was assigned an individual column, and the rows corresponded to responses from various respondents, stratified by age group, income, race/ethnicity, and other demographic characteristics.

SJS, KM, and MN coded the responses until no new information remained (theoretical saturation was attained). The decision to determine whether saturation had been attained was reached in consensus after each reviewer had independently reviewed their own notes. After reaching saturation, the evaluators independently reviewed the codes and compared the general responses to responses stratified by race/ethnicity, disability, and household income to note of any thematic differences between groups. Even after reaching saturation in the overall dataset, each evaluator independently analyzed the matrix by demographics and across all cases to identify dominant themes between and within groups, and the team derived the final themes by consensus.

It is important to acknowledge the positionality of the authors. While this study focused on the experiences of BIPOC communities and female parents, none of the authors self-identify as Black or Indigenous. One of the authors identifies as a mother of young children.

## Results

In 2021, 7033 parents and caregivers participated in the survey: 62% online and 31% by phone, and 85% of responses were in English (Table 1). As measured by child race/ethnicity, participation in the survey represented the racial and ethnic diversity of King County children (CHAT, 2021). Out of 7033 total respondents, 6593 respondents completed open-ended responses, with 5341 describing impacts on both the child and family.

**Table 1:** Demographic distribution of BSKHS participants who responded to open-ended questions about the impact of COVID-19

Age	Child’s age		5.8
	Respondent’s age		38.7
Child’s gender	Female		3197 (49.3%)
	Male		3223 (49.7%)
	Trans and/or nonbinary		61 (0.9%)
Respondent’s gender	Female		5179 (79.9%)
	Male		1260 (19.4%)
	Trans and/or nonbinary		41 (0.6%)
Child’s race*	American Indian/Alaskan Native	Total	217 (3.5%)
	Asian	Total	1965 (31.7%)
		Indian	409 (6.6%)
		Chinese	636 (10.2%)
		Filipino	304 (4.9%)
		Japanese	209 (3.4%)
		Korean	172 (2.8%)
		Vietnamese	289 (4.7%)
		Cambodian	60 (1.0%)
		Thai	32 (0.5%)
		Taiwanese	52 (0.8%)
		Another South Asian group	38 (0.6%)
		Another Southeast Asian group	60 (1.0%)
		Another Asian group	60 (1.0%)
	Black/African American	Total	945 (15.2%)
		Black/African American	647 (10.4%)
		Somali	119 (1.9%)
		Ethiopian	113 (1.8%)
		Another East African group	55 (0.9%)
		Another Black or African American group	129 (2.1%)
	Hispanic or Latina/o/x	Total	884 (14.2%)
		Mexican or Mexican American	735 (11.8%)
		Cuban, Puerto Rican, or Dominican	70 (1.1%)
		Salvadoran	60 (1.0%)
		Guatemalan	45 (0.7%)
		Another Central American group	60 (1.0%)
		South American	113 (1.8%)
		Another Hispanic or Latina/o/x group	102 (1.6%)
	Native Hawaiian/Pacific Islander	Total	150 (2.4%)
		Native Hawaiian	41 (0.7%)
		Samoan	52 (0.8%)
		Another Pacific Islander group	65 (1.0%)
	White	Total	3667 (59.1%)
		Middle Eastern or North African	155 (2.5%)
		White	3562 (57.4%)
	Another race	Total	35 (0.6%)
Household income	Less than $25,000		644 (10.6%)
	$25,000 to $49,999		939 (15.5%)
	$50,000 to $99,999		1104 (18.3%)
	$100,000 to $199,999		1815 (30.0%)
	$200,000 or more		1545 (25.5%)
Survey response language (N = 7,033 total responses)	Chinese		220 (3.1%)
	English		5990 (85.2%)
	Russian		66 (0.9%)
	Somali		40 (0.6%)
	Spanish		537 (7.6%)
	Vietnamese		159 (2.3%)
Proportion of children with a self-reported developmental delay**			9.6%

*Race and gender groups are not mutually exclusive, so add to > 100%

**Weighted result (unweighted estimate is 9.1%)

### Health and Emotional Well Being

Parents shared that lack of in-person socialization, inability to access schooling, and isolation and quarantine during the COVID-19 pandemic contributed to unmet social-emotional and mental health needs for school-aged children and delayed milestones for infants and toddlers. Many parents worried about limited opportunities for infants and toddlers to socialize outside their household:

“*Honestly, the COVID pandemic has really affected [my child]. Since lockdown started when he was an infant, he still has had barely any exposure to the outside or other people/kids in general. It has made him act nervous and insecure in any other environment besides our house.”*—Mother of Black/African American 0–5-year-old

Some parents said children experienced heightened anxiety around new people and places due to lack of socialization, while other children experienced regressions in developmental milestones or their ability to manage developmentally-appropriate responses.

There were also examples of resilience among children, including examples of older children becoming more self-sufficient with homework and technology, engagement with school, and deeper connections between siblings.

“*[My child] has stepped up well to keep engaged in school and with local neighborhood friends. He is emotionally, physically, and developmentally healthy. However, COVID has taken a toll on his joy.”*—Father of white K-5th grader

The pandemic also exacted a heavy toll on the health and emotional well-being of parents. Parents were exhausted by the competing demands of childcare and remote schooling in the context of broader social isolation and extended time with immediate family. This was particularly true for mothers, single parents, and families with children living with disabilities. The stress of worrying about contracting COVID or having family members fall ill and pass away only added to the anxiety.

“*I have been working at home with 5 kids for a year. Trying to manage 5 online schedules alone is really hard. I miss friends. I am drinking more and have started therapy to deal with the new normal.”*—Mother of Black/African American K-5th grader

### Childcare

Many families reported disruptions or changes to childcare. Given that many families already struggled to find affordable, accessible childcare in King County, this frequently made a challenging situation even more difficult (Department of Commerce, 2019).

*“Have problems finding child sitters, even with family, when one or more parents have to work. Parents sometimes have to leave work to support watching children.”*—Mother of Black/African American K-5th grader

Families also reported an inability to rely on support from grandparents or other extended family due to the pandemic. In some cases, respondents mentioned not wanting to risk exposing older family members to COVID, while in others, they cited travel restrictions as a barrier.

*“I have struggled with finding a trusted nanny that is affordable while I work because my parents are unable to cross the border at this time to do help with this task.”*—Mother of Latina/x/o 0–5-year-old

Some families removed their child from childcare due to worries about COVID, or simply chose not to seek an alternative arrangement when their primary source of childcare closed. Parents also experienced stress and exhaustion trying to balance work demands (including while working from home), childcare, and online schooling:

*“Since the parents need to work more and in different ways and our access to in person school and childcare has been extremely limited, the children are getting less quality and more screen time and many daily household tasks are just too much and falling to the wayside.”*—Mother of white 0–5-year-old child living with a disability

Some families reported that new childcare demands prevented them from working or attending to other needs. In some cases, this led to lost income through reduced work hours or job losses.

*“One of us needs to take care of [child] at home instead of going to work.”*—Mother of Asian 0–5-year-old with a household income of $50,000-99,000

### Elementary Schooling

Many parents expressed that online learning was difficult for their child, particularly preschoolers and young children, and it was challenging for parents to support children appropriately while juggling work and other childcare demands. A few parents mentioned they were unable to support their child’s remote learning since they did not know the information themselves.

*“Our 2nd grader is not engaged with learning, and we don’t have time to fully commit to helping him. We do the minimum with assignments and Zoom school, but nothing extra. We don’t have the energy or time. It has been extremely hard to balance work and Zoom school and we worry about his education—mostly reading and his lack of attachment to his teachers and friends."*—Mother of Native Hawaiian Pacific Islander K-5th grader

Several parents mentioned their child fell behind during remote learning. Parents also noted that their child’s relationship with school had changed, and their child no longer enjoyed school. Additionally, parents expressed that children who were English language learners or who experienced speech difficulties struggled to participate remotely.

*“[Child] has difficulty with speech. It is difficult for him to fully participate in his class activities and be understood by others*.”—Mother of Black/African American K-5th grade child living with a disability

Importantly, some parents felt their child was thriving in the online learning environment, or that they preferred the online educational experience. Among parents who shared positive experiences with remote learning, parents were more likely to have African American and NHPI children, yet it is important to note that more BIPOC parents overall shared negative experiences with online schooling. A few parents mentioned that online schooling allowed their child to get away from bullying, racism, or other unsafe school environments, while others appreciated having additional insight into their child’s schooling.

*“Happier spending time with family and connecting with his culture and not experiencing racial bias at school.”*—Father of Black/African American K-5th grader

### Family Relationships

Parents frequently mentioned how their family dynamics and relationships had been impacted by the pandemic. Many parents expressed gratitude for more quality time with family and improved sibling relationships, but also commented that this additional time together came with challenges like bickering and less personal time.

“*[It is] positive [that] family is more united; we depend more on each other and speak with family more. Negative not enough space from each other, can get frustrated with each other.*”—Father of Latina/x/o K-5th grader

Parents missed seeing their extended family and community and lamented the lack of opportunities to build relationships with family, especially among immigrant families. Some expressed that it was particularly difficult to not be able to gather as a community during the pandemic, and Native Hawaiian and Pacific Islander (NHPI) families particularly expressed a loss of cultural connection as a result. This is consistent with NHPI communities’ stated needs to center family and community in pandemic response (Kamaka ML et al., 2021) as well as the success of culturally grounded health interventions for NHPI people that incorporate family and community orientation (Kaholokula JK et al., 2018).

“*In our culture, we stay and get together, and we don’t have that luxury anymore. We don’t see our people anymore*."—Father of Native Hawaiian Pacific Islander 0–5-year-old

Findings revealed a disproportionate impact on mothers, with many sharing that they left employment to provide childcare and were bearing the brunt of additional responsibilities at home. Several mothers shared that these additional responsibilities left them exhausted and had negatively impacted their mental health.

“*Well, lots of time spent together and so tension between myself and my partner. I expect more hands-on help with the kids and the house, but he seems unwilling to do so. We talk about it all the time, but not much has changed.*”—Mother of Native Hawaiian Pacific Islander K-5th grader with a household income <$50,000

### Disproportionate Impacts on Children Living with Disabilities, BIPOC Communities, Low-Income Families, and Mothers or Female Caregivers

While these themes were broadly observed across all demographics, the impact of the pandemic was not equally shared. Financial struggles, job losses, and limited work-life balance were especially pronounced among BIPOC communities, with many parents reporting that they relied on government programs like the Special Supplemental Nutrition Program for Women, Infants, and Children (WIC) and community-based resources or non-profits for help to make it through the pandemic.

Parents with young children living with disabilities struggled to access resources and worried about regressions and impacts on speech development and social-emotional learning. Accessing therapy and other specialized services was especially difficult during this time, and remote learning only made things worse for children living with disabilities.

*“[My child] has an IEP and remote learning has really negatively impacted her progress with learning. She needs one-on-one assistance. With both of us being full-time working parents, it has been difficult providing [my child] with the best learning support she needs.”*—Mother of Latina/x/o K-5th grader living with a disability

While some African American and Latina/x/o families adapted well to online learning, many said that online school does not work well for young kids:

“S*maller children need constant structure.”*—Father of Black/African American and Latina/x/o 0–5-year-old

Childcare disruptions disproportionately impacted mothers who often had to stop working to provide childcare, which was especially true among low-income women and Latinas.

“*There’s also more tension in our marriage about who is doing what in terms of childcare vs. work time. The boundaries are less clear when I am also home, because originally my spouse was supposed to be full time care provider while I worked …We’ve also had a lot of stress-emotionally-in not being able to see our family for such long stretches of time.”*—Parent of 0–5-year-old

## Conclusions for Practice

This study describes the impacts of the COVID pandemic on young children and families, while focusing explicitly on the experiences of BIPOC families and mothers of all races. To our knowledge, the use of open-ended questions on a mixed-mode, multi-lingual, population-based survey is unique and richly describes impacts of the pandemic using parent/caregiver generated themes. Key findings include that the COVID pandemic disrupted access to childcare, limited socialization opportunities for children and families, and increased feelings of stress, anxiety, and other adverse mental health outcomes, which are beginning to be documented (Ali et al., 2021; Bullinger et al., 2021; Del Boca et al., 2020; Sojli et al., 2021). The effects of the pandemic were disproportionately and more acutely felt by BIPOC communities, families who had children with disabilities, mothers and other primary caregivers, and low-income households, which has been documented elsewhere in the literature as well (Tai et al., 2021; Lebrasseur et al., 2021; Houtrow et al., 2020; Harris et al., 2021). Parents struggled to access resources for young children living with disabilities, and worried about impacts on speech development and social-emotional learning (Houtrow et al., 2020). The stress and feelings of isolation brought on by the pandemic increased mental health concerns, such as depression and anxiety, across the age spectrum. Children and their families will need ongoing support to address changes in social-emotional development and mental health, especially those who could not access needed services during the pandemic.

The study had a few limitations: data was collected using a survey and parents or interviewers wrote in responses from families, so there may be nuances that are not reflected. These results were not vetted by parents in the community, and future work might include member-checking (validating the final themes through a second round of discussions with community participants) to ensure these themes resonate with families (Jung et al., 2019; Birt et al., 2016). However, there were several strengths in this analysis: the mixed-methods approach provides relevant and timely data about the impacts of the COVID-19 pandemic on children and families in King County, WA, based on a large, racially and linguistically representative sample with enough power to examine population-specific themes. The results were also widely shared with diverse audiences, including various program teams and school-based taskforces, who shared that the themes resonated.

Given the concern parents reported about children’s social-emotional development and mental health, systems that serve the developmental and emotional needs of children and young people will need to be scaled up (Araújo et al., 2021). Access to affordable, stable, and high-quality childcare was critical in supporting families and children. In many places, the demand for childcare and early learning far exceeds the capacity of current facilities, particularly for low-income families (Third Sector Intelligence Inc., 2018; State of the Children, 2021). For example, in King County, one in four children eligible for subsidized early learning did not have access to a slot even before the COVID-19 pandemic (Third Sector Intelligence Inc. 2018). Initiatives such as Best Starts for Kids that support a wide range of supports for health promotion, prevention, and early intervention throughout children’s development and “strengthen childcare as critical infrastructure” in King County are critical in meeting this gap (About Best Starts for Kids, 2022; Best Starts for Kids 2.0, 2022). Policies should be developed to ensure that families have access to stable and affordable childcare.

The pandemic impacted many families’ abilities to meet basic needs such as food and housing (Zuckerman et al., 2020). This study found that many families identified public programs as being essential to their ability to continue meeting these needs, and these programs should continue to be available as long as families need them.

Although many families had negative experiences with remote learning, some noted that remote learning better suited their child’s learning style. Remote learning could be incorporated into future hybrid educational options to mitigate COVID spread and meet the needs of students (Mauras et al., 2021).

Finally, we note that women have been disproportionately impacted by the stressors of COVID and the trade-off between employment and childcare. Many mothers had to stop working to provide childcare, particularly among low-income women and Latinas. For some families that included a mother and father, mothers took on the additional responsibilities of childcare and online schooling, even when both parents were working from home. Continued progress towards gender equity demands solutions across a wide range of domains from paid parental leave for parents of all genders to government-supported childcare (England et al., 2020).

In summary, we documented several impacts on parents/caregivers and young children in the 2020–2021 time period of the COVID-19 pandemic. The impacts of the COVID-19 pandemic will be long-lasting, and lessons learned can inform recovery activities and inform future pandemic responses. Across the domains of childcare, other basic needs, social-emotional learning, mental health, and access to services, partnerships between parents and public institutions will be best positioned to identify effective and culturally relevant strategies to mitigate pandemic impacts. The Best Starts for Kids levy provides one example of funding and partnerships to address these areas.

## Public Health Implications

- Profound changes in children’s social-emotional development and mental health may require additional supports to help children thrive.
- Parents appreciated more insight into what happens in the classroom, and some scholars thrived in an online learning environment.
- Government programs and financial assistance were important sources of support.
- Access to affordable and stable childcare were critical in supporting families and children.
- Mothers have been disproportionately impacted by the stressors of COVID and the trade-off between working and childcare.

## Data Availability

Data is available from the corresponding author upon reasonable request.

## References

[CR1] About Best Starts for Kids. (2022). King County Department of Community and Human Services. https://kingcounty.gov/depts/community-human-services/initiatives/best-starts-for-kids/background.aspx

[CR2] Ali U, Herbst CM, Makridis CA (2021). The impact of COVID-19 on the US child care market: Evidence from stay-at-home orders. Economics of Education Review.

[CR3] Araújo LAD, Veloso CF, Souza MDC, Azevedo JMCD, Tarro G (2021). The potential impact of the COVID-19 pandemic on child growth and development: A systematic review. Journal De Pediatria.

[CR4] Best Starts for Kids Health Survey. (2021). https://kingcounty.gov/depts/community-human-services/initiatives/best-starts-for-kids/survey.aspx#:~:text=The%20Best%20Starts%20for%20Kids%20Health%20Survey%20is,the%20University%20of%20Washington%20to%20collect%20this%20information.

[CR5] Birt L, Scott S, Walter F (2016). Member checking: A tool to enhance trustworthiness or merely a nod to validation?. Qualitative Health Research.

[CR6] Bullinger LR, Raissian KM, Feely M, Schneider WJ (2021). The neglected ones: Time at home during COVID-19 and child maltreatment. Children and Youth Services Review.

[CR8] Best Starts for Kids 2.0. (2022). King county department of community and human services. https://kingcounty.gov/elected/executive/constantine/initiatives/best-starts-for-kids.aspx.

[CR7] Del Boca D, Oggero N, Profeta P, Rossi M (2020). Women’s and men’s work, housework and childcare, before and during COVID-19. Review of Economics of the Household.

[CR9] Early Learning Facilities Development Proposal for King County and the Puget Sound Taxpayer Accountability Account. (2018). Third Sector Intelligence Inc. https://www.childcare.org/ckfinder/userfiles/files/Early%20Learning%20Facilities%20Development%20Proposal(1).pdf

[CR10] Early Support for Infants and Toddlers (ESIT). (2022). Department of Community & Human Services. https://kingcounty.gov/depts/community-human-services/developmental-disabilities/services/babies-toddlers.aspx.

[CR11] Economic, social, and overall health impacts dashboard. (2022). Public Health-Seattle & King County (PHSKC). https://kingcounty.gov/depts/health/covid-19/data/impacts.aspx

[CR12] England P, Levine A, Mishel E (2020). Progress toward gender equality in the United States has slowed or stalled. Proceedings of the National Academy of Sciences USA.

[CR13] Furfaro, H., O’Sullivan, J., & Bazzaz, D. (2021). Washington Gov. Inslee to require K-12 schools to offer some in-person learning as part of COVID-19 recovery. Seattle Times. https://www.seattletimes.com/seattle-news/education/gov-inslee-order-will-require-washingtons-k-12-schools-to-offer-some-in-person-learning-as-part-of-covid-19-recovery/

[CR14] Gale NK, Heath G, Cameron E, Rashid S, Redwood S (2013). Using the framework method for the analysis of qualitative data in multi-disciplinary health research. BMC Medical Research Methodology.

[CR15] Harris B, McClain MB, O’Leary S, Shahidullah JD (2021). Implications of COVID-19 on school services for children with disabilities: Opportunities for interagency collaboration. Journal of Developmental & Behavioral Pediatrics..

[CR16] Houtrow A, Harris D, Molinero A, Levin-Decanini T, Robichaud C (2020). Children with disabilities in the United States and the COVID-19 pandemic. Journal of Pediatric Rehabilitation Medicine.

[CR17] Impact of the COVID-19 Pandemic on Early Identification of Developmental Delays and Disabilities and Opportunities for Improvement. (2022). U.S. Centers for Disease Control and Prevention. https://www.cdc.gov/ncbddd/actearly/pdf/impact-covid-developdisabil-508.pdf.

[CR18] Jung H, Ro E (2019). Validating common experiences through focus group interaction. Journal of Pragmatics.

[CR19] Kaholokula JK, Ing CT, Look MA, Delafield R, Sinclair K (2018). Culturally responsive approaches to health promotion for Native Hawaiians and Pacific Islanders. Annals of Human Biology.

[CR20] Kamaka ML, Freitas SM, Marshall SM, Walsh ME, Kamakawiwo’ole S, Miller JM, Balaz K, Vakalahi H (2021). He ‘A’ali’i Kū Makani Mai Au: Developing a cultural framework for advancing COVID-19 related, community-informed health policies. Hawai’i Journal of Health & Social Welfare.

[CR21] Karpman, M., Zuckerman, S., Gonzalez, D., & Kenney, G. M. (2020). The COVID-19 pandemic is straining families’ abilities to afford basic needs. Washington, DC: Urban Institute. https://www.urban.org/research/publication/covid-19-pandemic-straining-families-abilities-afford-basic-needs

[CR22] King County School Health (School-Based Health Centers). (2022). Public Health-Seattle & King County (PHSKC). https://kingcounty.gov/depts/health/child-teen-health/school-health.aspx.

[CR23] Lebrasseur A, Fortin-Bédard N, Lettre J, Bussières EL, Best K, Boucher N, Hotton M, Beaulieu-Bonneau S, Mercier C, Lamontagne ME, Routhier F (2021). Impact of COVID-19 on people with physical disabilities: a rapid review. Disability and Health Journal.

[CR24] Mauras S, Cohen-Addad V, Duboc G, Dupré la Tour M, Frasca P, Mathieu C, Viennot L (2021). Mitigating COVID-19 outbreaks in workplaces and schools by hybrid telecommuting. PLoS Computational Biology.

[CR25] Meherali S, Punjani N, Louie-Poon S, Abdul Rahim K, Das JK, Salam RA, Lassi ZS (2021). Mental health of children and adolescents amidst COVID-19 and past pandemics: A rapid systematic review. International Journal of Environmental Research and Public Health.

[CR26] Public Health-Seattle & King County. (2020). Update for March 16, 2020: New State And Local Orders Issued To Protect Residents’ Health From COVID-19. Public Health Insider. https://publichealthinsider.com/2020/03/16/update-for-march-16-2020-new-state-and-local-orders-issued-to-protect-residents-health-from-covid-19/

[CR27] Race and ethnicity data dashboard. (2021). Public Health-Seattle & King County (PHSKC). https://kingcounty.gov/depts/health/covid-19/data/race-ethnicity.aspx.

[CR28] Schachter, A., Song, L., Neal, S., Pajimula, F., Johnson, K., Laurent, A., Kimball, E., Wong, E. (2020). Increases in Food Needs in King County, WA, Spring-Summer 2020. Public Health-Seattle & King County; Assessment Policy Development and Evaluation Unit

[CR29] Sojli E, Tham WW, Bryant R, McAleer M (2021). COVID-19 restrictions and age-specific mental health—US probability-based panel evidence. Translational Psychiatry.

[CR30] State of the children: Early learning & care. (2021). King County, Washington. https://washingtonstem.org/wp-content/uploads/2021/04/SoTC-King-County-4-8-21.pdf

[CR31] Tai DB, Shah A, Doubeni CA, Sia IG, Wieland ML (2021). The disproportionate impact of COVID-19 on racial and ethnic minorities in the United States. Clinical Infectious Diseases.

[CR32] The Mounting Costs of Child Care. (2019). Impacts of child care affordability and access to Washington’s employers and economy. Washington State Department of Commerce. http://www.commerce.wa.gov/wp-content/uploads/2019/09/MountingCostsReport_FINAL.pdf

[CR33] Washington State Office of Financial Management, Forecasting Division, single year intercensal estimates 2001–2020, Community Health Assessment Tool (CHAT). (2021). https://doh.wa.gov/public-health-healthcare-providers/public-health-system-resources-and-services/community-health-assessment-and-improvement/chat

[CR34] Wong E, Moore K, Woodward D, Laurent AA. (2017). Novel Sampling Methodologies for a Local Population-Based Survey about Health and Wellbeing of Young Children in King County, Washington: The Best Starts for Kids Health Survey. https://cste.confex.com/cste/2017/webprogram/Paper8503.html.

